# An Investigation of Molecular Docking and Molecular Dynamic Simulation on Imidazopyridines as B-Raf Kinase Inhibitors

**DOI:** 10.3390/ijms161126026

**Published:** 2015-11-16

**Authors:** Huiding Xie, Yupeng Li, Fang Yu, Xiaoguang Xie, Kaixiong Qiu, Jijun Fu

**Affiliations:** 1 Department of Chemistry, Yunnan University, Kunming 650091, China; front701228.student@sina.com; 2 Department of Chemistry, School of Pharmaceutical Science & Yunnan Key Laboratory of Pharmacology for Natural Products, Kunming Medical University, Kunming 650500, China; liyupeng26@126.com (Y.L.); yufang519@163.com (F.Y.); Fujj920@163.com (J.F.)

**Keywords:** imidazopyridine, B-Raf kinase, molecular docking, molecular dynamic simulation

## Abstract

In the recent cancer treatment, B-Raf kinase is one of key targets. Nowadays, a group of imidazopyridines as B-Raf kinase inhibitors have been reported. In order to investigate the interaction between this group of inhibitors and B-Raf kinase, molecular docking, molecular dynamic (MD) simulation and binding free energy (Δ*G*_bind_) calculation were performed in this work. Molecular docking was carried out to identify the key residues in the binding site, and MD simulations were performed to determine the detail binding mode. The results obtained from MD simulation reveal that the binding site is stable during the MD simulations, and some hydrogen bonds (H-bonds) in MD simulations are different from H-bonds in the docking mode. Based on the obtained MD trajectories, Δ*G*_bind_ was computed by using Molecular Mechanics Generalized Born Surface Area (MM-GBSA), and the obtained energies are consistent with the activities. An energetic analysis reveals that both electrostatic and van der Waals contributions are important to Δ*G*_bind_, and the unfavorable polar solvation contribution results in the instability of the inhibitor with the lowest activity. These results are expected to understand the binding between B-Raf and imidazopyridines and provide some useful information to design potential B-Raf inhibitors.

## 1. Introduction

In spite of the progress in medicine, developing new anticancer drugs is still important because cancer still acts as a major problem of health all over the world [1]. According to the recent reports [2,3,4], MAPK pathway (also called Ras–Raf–MEK–ERK pathway) is very crucial for cell survival and proliferation because this pathway can be activated easily in human cancers (up to 30%). There are three isoforms (A-Raf, B-Raf, and C-Raf) for Raf kinase in this pathway [5], and because the mutations of B-Raf kinase in human cancers is up to 7%, they has been considered as the primary activator in this pathway. There is a different mutation frequency of B-Raf kinase in various human cancers, such as colorectal cancers (10%), thyroid cancers (30%), ovarian cancers (35%), and melanoma (50%–70%) [6]. Thus, B-Raf kinase has been a key target in recent cancer treatment [7,8,9].

**Figure 1:**
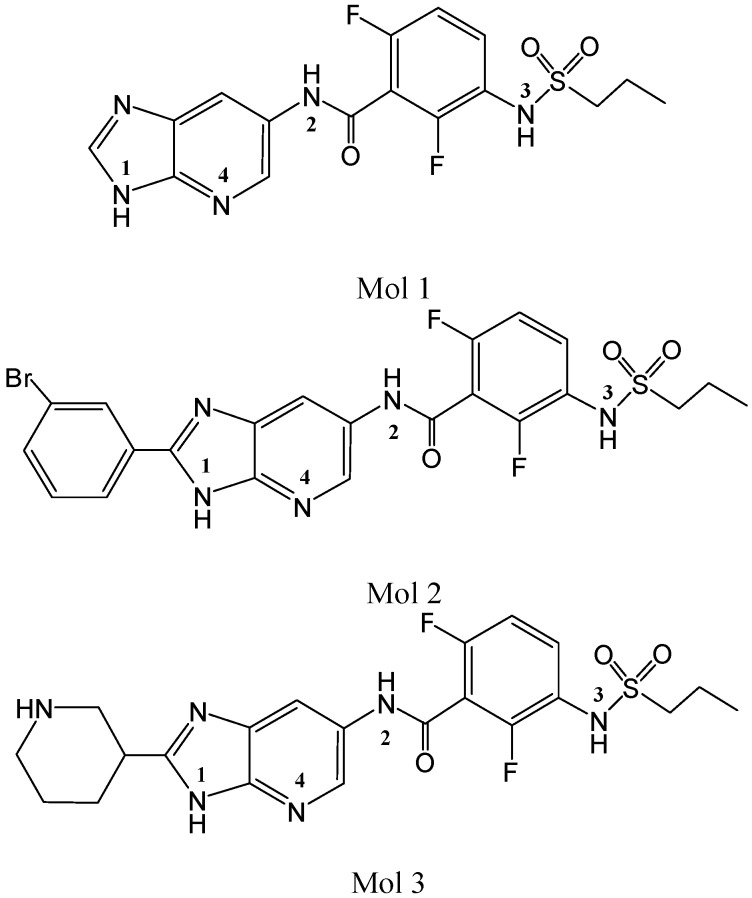
Mol 1, Mol 2, and Mol 3 structures.

The first B-Raf kinase inhibitor (BRI) approved by the Food and Drug Administration (FDA) is Sorafenib, which is used to treat hepatocellular carcinoma and renal cell carcinoma in clinic. Vemurafenib is the second BRI approved by FDA, which is used to treat metastatic melanoma in clinic. Furthermore, some BRIs are in various stage of clinical development, such as RAF265, GSK2118436, and SB-590885. In spite of the success of clinical efficiency of these inhibitors in cancer treatments, they still have some major side effects and can develop drug resistance. Therefore, it is still important to develop other potent and selective BRIs [10]. Recently, Newhouse *et al.* have synthesized a series of imidazopyridines as BRIs, which show excellent potency and selectivity. These BRIs bind in a DFG-in, αC-helix out, inactive conformation of wild-type B-Raf kinase [11]. In our previous work, we performed 3D QSAR, pharmacophore modeling, and virtual screening studies on this series of molecules to help design more potential BRIs [12]. In order to know the interaction between this series of inhibitors and B-Raf kinase, an investigation of molecular docking, MD simulation and Δ*G*_bind_ calculation on this kind of BRIs were carried out in this work, in which three inhibitors (Mol 1, Mol 2, and Mol 3) were selected (Figure 1). The reason we chose these three molecules is that the crystal structure of B-Raf kinase combined with Mol 1 is available, Mol 2 shows the highest inhibitory activity, and Mol 3 shows the lowest activity. Their activities (IC_50_) are 61 nM, 0.76 nM, and 167 nM, respectively [11].

In present work, molecular docking was carried out between all the imidazopyridines and B-Raf kinase. MD simulations were performed between the three inhibitors (Mol 1, Mol 2 and Mol 3) and B-Raf kinase, and the analysis of hydrogen bond (H-bond) in MD simulations was performed as well. Furthermore, the Δ*G*_bind_ was computed by MM-GBSA method based on MD trajectories, and the contributions to the Δ*G*_bind_ were also analyzed.

## 2. Results and Discussion

### 2.1. Molecular Docking

**Figure 2:**
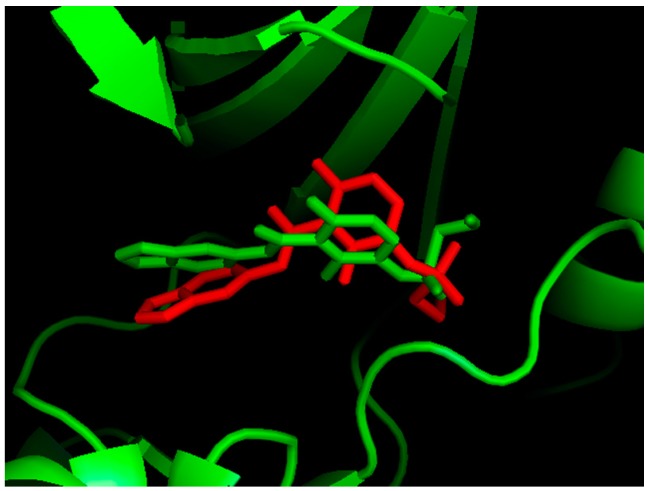
Comparison between the docked and X-ray crystal structures of Mol 1 (red: docked structure; green: crystal structure).

**Figure 3:**
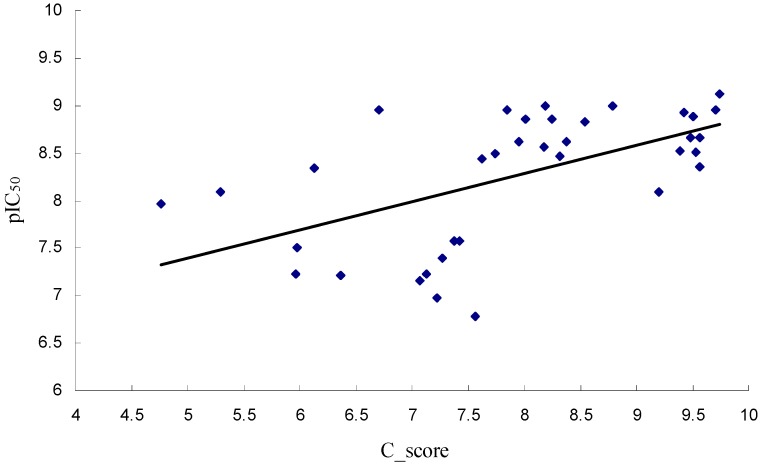
The correlation between C_score values and pIC_50_ values of 36 imidazopyridines.

To validate the docking method and docking accuracy, Mol 1, was docked into the binding site of B-Raf kinase receptor. Both Mol 1 ligand and B-Raf kinase receptor were isolated from the complex crystal structure (PDB code: 4MBJ) [11]. The root-mean-square deviation (RMSD) between the docked and the crystal structures of Mol 1 was only 1.698 Å (less than 2 Å), which is satisfactory. Figure 2 shows that the docked structure (red color) and the X-ray crystal structure (green color) are quite similar. In addition, all the 36 imidazopyridines were docked into the binding pocket of B-Raf kinase receptor successfully. The chemical structures, biological activity values and docking C_scores of the imidazopyridines are shown in Table S1 (supplementary data). Almost all inhibitors show high C_score values, which are more than 5.0. The correlation between C_score values and biological activity (pIC_50_ values) of 36 imidazopyridines is shown in Figure 3. The above results indicate an acceptable reliability of the docking method for the B-Raf kinase receptor and these inhibitors.

**Figure 4:**
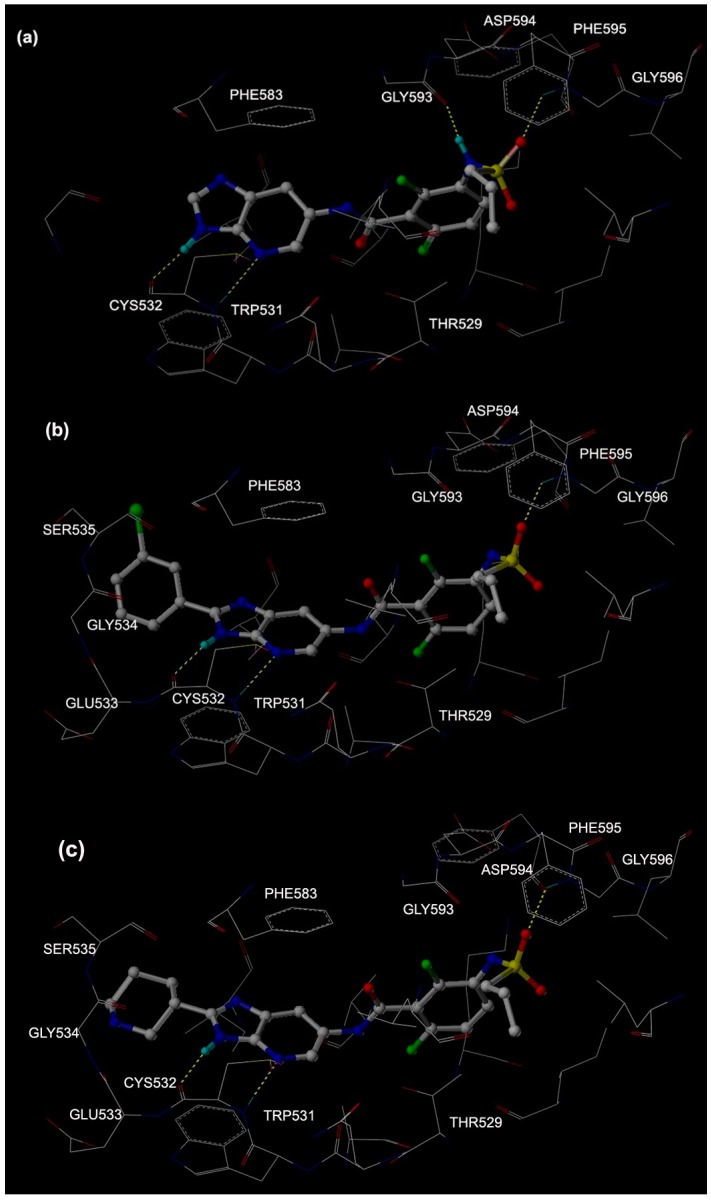
Docking modes between B-Raf kinase and Mol 1 (**a**), Mol 2 (**b**) and Mol 3 (**c**).

In order to illustrate the interactions between B-Raf kinase and imidazopyridine, we focus on receptor-ligand interactions between B-Raf kinase and Mol 1 (the ligand of 4MBJ), Mol 2 (the most active inhibitor), and Mol 3 (the least active inhibitor). Figure 4a shows the docking mode between B-Raf kinase and Mol 1, in which four H-bonds were formed: the first one is between the >C=O of CYS 532 and the H-N1 (Figure 1) of Mol 1 (>C=O···H–N1) with a distance of 1.89 Å and a deviated angle of 15.9°; the second one is formed by amide hydrogen of TRP 531 and N4 of Mol 1 (–N–H···N4, 2.20 Å, 18.2°); the third one is between the carbonyl oxygen of GLY 593 and H-N3 of Mol 1 (>C=O···H–N3, 2.13 Å, 45.0°); and the fourth one is between the amide hydrogen of PHE 595 and sulphuryl oxygen atom of Mol 1 (–N–H···O=S, 2.04 Å, 54.8°). Due to their large angles (45.0° and 54.8°), the third and fourth H-bonds show less stability than the first and second H-bonds. As shown in Figure 4a, there are a π–π stacking contact between the imidazolepyridine ring of Mol 1 and the aromatic ring of PHE583, and a hydrophobic interaction between the benzene ring of Mol 1 and the side chain of THR529. The above observations are consistent with the previous studies [13].

The docking mode between B-Raf kinase and Mol 2 can be seen in Figure 4b, in which three H-bonds were formed: the first one is between the >C=O of CYS 532 and the H–N1 of Mol 2 (>C=O···H–N1, 1.96 Å, 10.3°); the second one is between the amide hydrogen of TRP 531 and N4 of Mol 2 (–N–H···N4, 2.18 Å, 9.7°); the third one is between the amide hydrogen of PHE 595 and sulphuryl oxygen atom (–N–H···O=S, 2.00 Å, 53.7°). The angle data of H-bonds indicates that the first two H-bonds are more stable than the third one. Similar with Mol 1, Mol 2 also shows a π–π stacking contact with aromatic ring of PHE583 and a hydrophobic interaction with the methyl group of THR529. Furthermore, the bromo–phenyl group attached the imidazole ring of Mol 2 interacts with the hydrophobic pocket formed by residues GLU533, Gly534, and SER535.

The docking mode between B-Raf kinase and Mol 3 is quite similar with the docking mode of Mol 2, which is shown in Figure 4c. The distance and angle of three H-bonds are as following: (>C=O···H–1, 1.93 Å, 10.8°); (–N–H···N4, 2.15 Å, 8.5°); (–N–H···O=S, 2.03 Å, 51.1°). The piperidine group connected with imidazole ring of Mol 3 is interacted with the hydrophobic pocket formed by residues GLU533, Gly534, and SER535.

As shown in Table S1, the pIC_50_ values of Mol 1, Mol 2, and Mol 3 are 7.215, 9.119, and 6.777, respectively, which means the inhibitory activity: Mol 2 > Mol 1 > Mol 3. However, the docking C_score values of them are 6.37, 9.74, and 7.56, respectively, which indicates the docking effect: Mol 2 > Mol 3 > Mol 1. The docking result is not in accordance with the inhibitory activity completely. Therefore, in order to further explore interactions between the B-Raf kinase and imidazolepyridines, MD simulations were carried out in the subsequent work.

### 2.2. MD Simulations

#### 2.2.1. MD Simulations Features

Although docking analysis can provide an acceptable binding mode, the solvent effect and flexibility of protein were not fully taken into account. Therefore, MD simulations were carried out on the three docked complexes (Mol 1, Mol 2, and Mol 3 complex) to further explore the ligand-receptor interactions.

**Figure 5:**
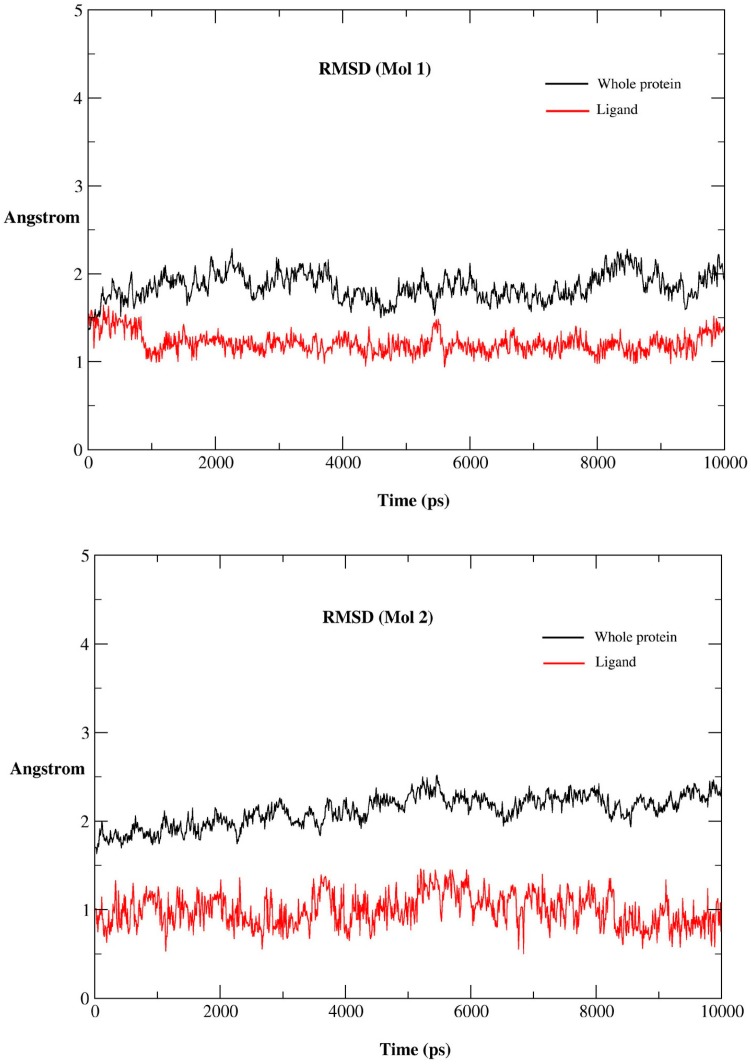

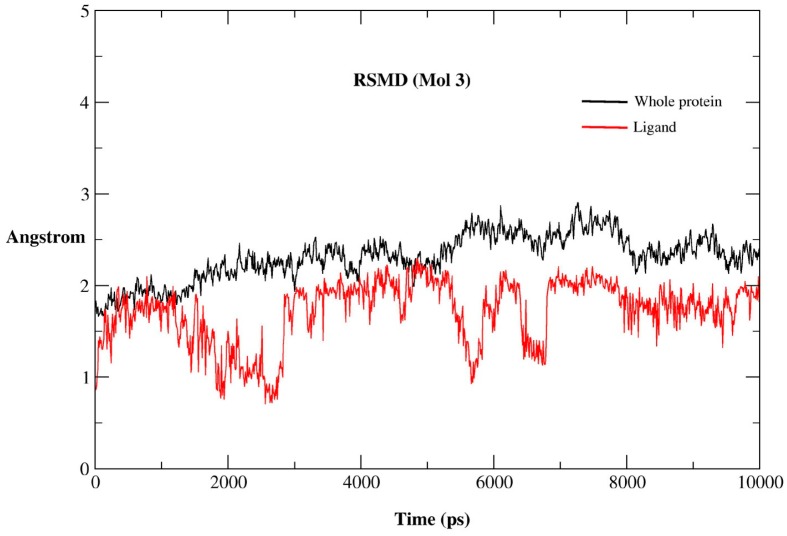
RMSD *versus* time for the Mol 1 complex, Mol 2 complex, and Mol 3 complex.

In order to evaluate the stability of the MD simulations, the properties of each complex (such as temperature, pressure, energy, and structure) were inspected during the entire MD trajectory. The fluctuations of temperature, pressure, and potential energy during the MD simulations are depicted in Figures S1–S3, respectively (supplementary data), which show that all of them are stable in the whole MD simulations process. The RMSD values of backbone atoms referring to the starting structure were used to monitor the dynamic stability of the MD trajectories. Figure 5 shows the RMSD for the Mol 1 complex, Mol 2 complex, and Mol 3 complex. For Mol 1 complex, the average RMSD fluctuations for the protein and ligand are 1.85 and 1.21 Å, respectively. The protein and Mol 1 reach to equilibrium after 4 ns. For Mol 2 complex, the protein and Mol 2 are quite stable after 6 ns, and the average RMSD fluctuations for protein and ligand are 2.12 and 0.99 Å, respectively. For Mol 3 complex, the protein, and Mol 3 reach to equilibrium after 8 ns, and the average RMSD fluctuations for the protein and ligand are up to 2.31 and 1.71 Å, respectively. The above results reveal the average RMSD fluctuations of the three ligands: Mol 2 < Mol 1 < Mol 3, which is in accordance with their inhibitory activity: Mol 2 > Mol 1 > Mol 3.

#### 2.2.2. RMSF for Residues of the Binding Pocket

To explore the stability of the binding pocket during the MD simulations process, the root-mean-squared fluctuations (RMSF) of all the residues around the ligand at a ≤5 Å distance were calculated by the VMD software. Before the RMSF calculation, the average structures of the complexes were computed within the last 1 ns trajectory of MD simulations, and then each residue surrounding the ligand was aligned to the average structure. The residues around the ligand and their RMSF values compared with the starting structures are listed in Table 1. In all the complexes, the RMSF for each residue surrounding the ligand is lower than 1.0 Å, which means that the binding pocket is quite stable during the MD simulation.

**Table 1:** Residues of the binding pocket and their RMSF values (Å).

Residues	Mol 1	Mol 2	Mol 3
ILE 463	0.075	0.109	0.127
PHE 468	0.081	0.148	0.207
VAL 471	0.275	0.194	0.265
ALA 481	0.614	0.701	0.704
LYS 483	0.452	0.512	0.563
LEU 505	0.540	0.201	0.805
LEU 514	0.621	0.573	0.597
ILE 527	0.678	0.712	0.597
THR 529	0.420	0.343	0.413
GLN 530	0.166	0.148	0.452
TRP 531	0.663	0.215	0.266
CYS 532	0.245	0.328	0.442
GLU 533	0.232	0.163	0.141
GLY 534	0.712	0.330	0.203
SER 535	0.158	0.244	0.217
PHE 583	0.653	0.746	0.958
GLY 593	0.177	0.248	0.512
ASP 594	0.197	0.241	0.218
PHE 595	0.086	0.098	0.064
GLY 596	0.835	0.566	0.748
LEU 597	0.405	0.610	0.604

#### 2.2.3. H-Bonds in MD Simulations

H-bonds interaction is quite important in the binding between receptor and ligand. In our work, H-bonds were computed within the last 1 ns trajectory of MD simulations, and all the possible hydrogen acceptors were taken into consideration, such as ligands, protein, and water molecules. The distance cutoff was set to 3.00 Å (<3.00 Å), the angle cutoff (deviation from linearity) was set to 60.00 degree (<60.00°), and the occupancy was set to 0.05% (>0.05%). The results of H-bonds analysis for the three systems in MD simulations are listed in Table 2.

**Table 2:** H-bonds analysis for Mol 1, Mol 2, and Mol 3 in MD simulations.

Complex	Acceptor	Donor	Occupancy (%)	Distance (Å)	Angle (°)
Mol 1	CYS 532 C=O	Lig N1–H	54.00	2.89 ± 0.07	18.51 ± 12.41
	THR 529 C=O	Lig N2–H	41.00	2.90 ± 0.07	20.54 ± 10.55
	ASP 594 C=O	Lig N3–H	48.00	2.82 ± 0.07	19.96 ± 10.74
	H_2_O	Lig N3–H	36.00	2.86 ± 0.10	20.48 ± 9.44
Mol 2	CYS 532 C=O	Lig N1–H	66.00	2.84 ± 0.09	26.11 ± 13.57
	THR 529 C=O	Lig N2–H	11.00	2.90 ± 0.05	23.84 ± 12.42
	ASP 594 C=O	Lig N3–H	69.00	2.81 ± 0.10	19.95 ± 10.67
Mol 3	CYS 532 C=O	Lig N1–H	10.00	2.91 ± 0.07	19.87 ± 11.91
	ASP 594 C=O	Lig N3–H	97.00	2.77 ± 0.08	14.26 ± 8.55
	Lig S=O	LYS 601 N–H1	42.50	2.82 ± 0.08	21.22 ± 11.35
	Lig S=O	LYS 601 N–H2	26.00	2.82 ± 0.08	22.81 ± 11.08

During the MD simulations, there were four hydrogen bonds formed in Mol 1 complex. The first H-bond is formed by the >C=O of CYS 532 and the H–N1 of Mol 1 with an occupation time of 54%, which is in accordance with the docking result. The second one is formed by the >C=O of THR 529 and the H–N2 of Mol 1 with an occupation time of 41%, the third one is formed by the >C=O of ASP 594 and the H–N3 of Mol 1 with an occupation time of 48%, and the fourth one is between oxygen of water (solvent) molecules and the H–N3 of Mol 1 with an occupation time of 36%. The second, third, and fourth H-bonds formed in MD are different from the docking result, which is caused by the solvent effect and movement of both receptor and ligand during the MD process.

Three hydrogen bonds were formed in the Mol 2 complex during the MD process. The first one is formed by the >C=O of CYS 532 and the H–N1 of Mol 2 (occupancy 66%), which is consistent with the docking result. The second one is formed by the >C=O of THR 529 and the H–N2 of Mol 2 (occupancy 11%), which indicates this H-bond is not important. The third one is formed by the >C=O of ASP 594 and the H–N3 of Mol 2 (occupancy 69%). The second and third H-bonds formed in MD show a difference with the docking result because of the movement of both receptor and ligand during the MD process.

Four hydrogen bonds were formed in Mol 3 complex during the MD process. The first one is formed by the >C=O of CYS 532 and the H–N1 of Mol 3 (occupancy 10%), which means this H-bond is not important. The second one is formed by the >C=O of ASP 594 and the H–N3 of Mol 2 (occupancy 97%), which means this H-bond is very important. The third and fourth H-bonds are between two amino hydrogen atoms of LYS 601 and the sulphuryl oxygen atom of Mol 3 (occupancy 42.5% and 26%). The H-bonds formed in MD are quite different from the docking result for Mol 3 complex, which indicates that there was a large movement of receptor and ligand during the MD process. It is in accordance with the result of RMSD fluctuations for Mol 3 complex.

### 2.3. Binding Free Energies

The calculated Δ*G*_bind_ of the three complexes are shown in Table 3. It can be seen that the calculated Δ*G*_bind_ values of the three complexes are consistent with their activities; their Δ*G*_bind_ values being: Δ*G*_bind_ (Mol 2) < Δ*G*_bind_ (Mol 1) < Δ*G*_bind_ (Mol 3), and their inhibitory activity pIC_50_ values being: pIC_50_ (Mol 2) > pIC_50_ (Mol 1) > pIC_50_ (Mol 3).

**Table 3:** Binding free energy (kcal·mol^−1^) for the three complexes.

Energy/Activity	Mol 1 Complex	Mol 2 Complex	Mol 3 Complex
Δ*E*_vdw_	−52.94	−59.02	−56.25
Δ*E*_ele_	−45.71	−46.95	−48.48
Δ*E*_gas_	−98.65	−105.97	−104.73
Δ*G*_GB_	53.66	56.45	61.94
Δ*G*_SA_	−6.61	−6.97	−7.11
Δ*G*_sol_	47.05	49.48	54.83
Δ*G*_bind_	−51.60	−56.49	−49.90
IC_50_	61 (nM)	0.76 (nM)	167 (nM)
pIC_50_	7.215	9.119	6.777

Δ*E*_gas_: molecular mechanics energy in gas phase; Δ*E*_ele_: electrostatic energy; Δ*E*_vdw_: van der Waals potential energy; Δ*G*_sol_: solvation free energy; Δ*G*_GB_: polar salvation free energy; Δ*G*_SA_: non-polar solvation free energy; Δ*G*_bind_: binding free energy; IC_50_: half maximal inhibitory concentration; pIC_50_: −logIC_50_.

The detailed contributions to the Δ*G*_bind_ also can be obtained from Table 3. Firstly, the van der Waals interaction contribution (Δ*E*_vdw_) is the most important to the Δ*G*_bind_ for each complex. For the Mol 2 complex, two benzene rings, and one imidazolepyridine ring show hydrophobic interaction with residues surrounding Mol 2, which is in accordance with the highest Δ*E*_vdw_ (−59.02 kcal·mol^−1^). For the Mol 3 complex, one benzene ring, one imidazole ring, and one piperidine ring show hydrophobic interaction with residues surrounding Mol 3, which is consistent with its middle Δ*E*_vdw_ (−56.25 kcal·mol^−1^). For the Mol 1 complex, one benzene ring and one imidazole ring show hydrophobic interaction with residues surrounding Mol 1, which accords with the lowest Δ*E*_vdw_ (−52.94 kcal·mol^−1^). The result also indicates that the bromo-phenyl group in Mol 2 has stronger hydrophobic interaction with the residues GLU533, Gly534, and SER535 than piperidine group in Mol 3 does. Secondly, the electrostatic contribution (Δ*E*_ele_) is important to the Δ*G*_bind_ for each complex as well, which is in accordance with the results of MD simulations because all three ligands formed no less than three H-bonds with the surrounding residues. Thirdly, the unfavorable polar solvation contribution (Δ*G*_GB_) affects the Δ*G*_bind_ greatly, which shows the main differences for the three complexes. The reason why Mol 3 shows the lowest activity and lowest stability is that it has the highest unfavorable polar solvation contribution (Δ*G*_GB_ = 61.94), which is more than Mol 1 (Δ*G*_GB_ = 53.66) and Mol 2 (Δ*G*_GB_ = 56.45). Finally, the favorable non-polar solvation contributions (Δ*G*_SA_) to the Δ*G*_bind_ for each complex are similar.

## 3. Experimental Section

### 3.1. Preparation of Protein and Ligands

The SYBYL 7.3 software (Tripos Inc., St. Louis, MO, USA) package installed on Linux workstations was used to prepare the protein and ligands. Among the imidazopyridines, the crystal structure of B-Raf kinase combined with Mol 1 can be obtained from the protein data bank (PDB code: 4MBJ) [11], so B-Raf kinase receptor and Mol 1 were isolated from the complex. The protein extracted from the complex was treated by removing all of the substructures, removing all of the water molecules and adding hydrogen atoms. Without any conformation change, Mol 1 isolated from the complex and hydrogen atoms were added and geometrically optimized with three steps: (i) optimization using Steepest Descent with Gasteiger-Marsili charges and Tripos force field; (ii) optimization using conjugate gradient; and (iii) optimization using BFGS [14]. The structures of all other imidazopyridines were built by modifying Mol 1, and geometrical optimizations were carried out by using the above procedure.

### 3.2. Molecular Docking

Molecular docking process between ligands and the receptor was carried out by using the Surflex-Dock module of SYBYL [15]. In this program, a computational representation of the intended binding site (ProtoMol) was used to dock ligands into the binding site of a receptor automatically [16]. In the present work, protomol_bloat was set to 0, protomol_threshold was set to 0.50 Å, and other parameters were set to default values. After docking, 10 conformations were present for each ligand, and the obtained final conformation was chose according to the following conditions: (i) the orientation of the docked conformation is in accordance with that of the ligand in crystal complex; and (ii) the conformation owns the highest C_score value. In the Surflex-Dock, the structures of ligands are flexible and the structure of the receptor is rigid.

### 3.3. MD Simulations

The AMBER 12 software package was used to carry out all the MD simulations [17]. The initial structures of Mol 1, Mol 2, and Mol 3 complexes for the MD simulations were obtained from the docked results. The FF12SB AMBER force field was taken in the protein, and charges were added to the protein by using the software database. The general AMBER force field (GAFF) was taken for ligands [18], and AM1-BCC method was applied to assign their partial charges because of the lack of partial charge parameters for ligands in GAFF force field [19]. The Antechamber suite in the AMBER 12 package was used to generate the topology files and atomic charges of ligands [20]. The Tleap module of the AMBER 12 was used to produce the topology and coordinate files of the whole system. The whole system was dipped into a water box of TIP3P with a margin distance of 10 Å [21]. In order to neutralize the charge of the system, a proper number of chloride ions were added. To deal with the long-range electrostatic interactions, the particle mesh Ewald (PME) was adopted during the MD simulations [22], and the cut-off distance of non-bonded interactions was set to 10 Å. The bonds involving hydrogen were constrained by the SHAKE algorithm [23].

Firstly, two stage energy minimizations were performed on each system: the algorithms (10,000 steps of the steepest descent and 10,000 steps of the conjugate gradient) with restrain were performed in the first stage; the same algorithms without restrain were further used in the second stage. Secondly, each system was heated from 0 to 300 K within 50 picoseconds (ps), gradually. Next, the system was equilibrated up to 500 ps at 300 K and constant pressure. Finally, a production process of 10 ns was performed in the constant temperature and pressure (NTP) with a step of 2 fs. The trajectories were recorded each 10 ps and the stability of the system was checked by the RMSD of the backbone. Trajectory analysis was carried out by using the CPPTRAJ [24].

### 3.4. Calculation of Binding Free Energy

The MM-GBSA method in AMBER 12 was used to compute the binding free energies (Δ*G*_bind_) of the receptor–ligand complexes [25]. All the 100 snapshots of the simulated structures within the last 1 ns trajectory of MD simulations were extracted to perform the Δ*G*_bind_ calculations. In MM-GBSA, Δ*G*_bind_ is calculated as follows:

(1) Δ*G*_bind_ = Δ*G*_complex_ − (Δ*G*_receptor_ + Δ*G*_ligand_)

where Δ*G*_complex_, Δ*G*_receptor_ and Δ*G*_ligand_ are the free energy of the complex, receptor, and ligand, respectively. They can be obtained by the following equations:

(2) Δ*G* = Δ*E*_gas_ + Δ*G*_sol_ − *T*Δ*S*_gas_

(3) Δ*E*_gas_ = Δ*E*_ele_ + Δ*E*_vdw_

(4) Δ*G*_sol_ = Δ*G*_GB_ + Δ*G*_SA_

where Δ*G* is free energy. *T*Δ*S*_gas_ represents entropy terms. The molecular mechanics energy in the gas phase (Δ*E*_gas_) consists of electrostatic interactions (Δ*E*_ele_) and van der Waals interactions (Δ*E*_vdw_). Solvation free energy (Δ*G*_sol_) is composed of polar contribution (Δ*G*_GB_) and the non-polar contribution (Δ*G*_SA_). Molecular Mechanics Poisson Boltzmann Born Surface Area (MM-PBSA) is the similar manner as MM-GBSA in calculating Δ*G*_bind_. However, for the calculation of electrostatic solvation energy, MM-PBSA uses the Poisson-Boltzmann model while MM-GBSA makes use of generalized Born model.

MM-PBSA calculation needs more time than MM-GBSA, and Hou T *et al.* reported that MM-GBSA shows better results than MM-PBSA in calculating relative Δ*G*_bind_ [26]. Therefore, MM-GBSA method was adopted to calculate the Δ*G*_bind_ in this work. Since the structures of three ligands are quite similar and the calculation time is limited, the entropy contribution was omitted in this study [27,28].

## 4. Conclusions

In present work, molecular docking, MD simulations and Δ*G*_bind_ calculation were performed. Some important residues in the binding pocket, such as CYS 532, TRP 531, GLY 593, ASP 594, THR529, PHE583, PHE 595, GLY596, GLU533, Gly534, and SER535, were identified by molecular docking. The results of molecular docking reveal that the binding modes of three inhibitors (Mol 1, Mol 2, and Mol 3) are similar. RMSD fluctuations of the three complexes were calculated during MD simulations, and the results are consistent with their inhibitory activities. RMSF values for each residue surrounding the ligand of the three complexes were also computed during MD simulations and each RMSF is lower than 1.0 Å, which indicates that the binding pocket is stable during the MD simulations. The H-bonds analysis reveals that some H-bonds in the MD simulations are different from H-bonds in the docking mode, which is caused by the movement of receptors and ligands during the MD process. The Δ*G*_bind_ obtained from MM-GBSA calculations reveals that the Mol 2 complex is the most stable, while the Mol 3 complex is the least stable, which are consistent with their inhibitory activities. By the contributions analysis to Δ*G*_bind_, both van der Waals and electrostatic contributions are significant to Δ*G*_bind_, and the main difference between Mol 1 and Mol 2 complexes, and the least stable Mol 3 complex, appears in the unfavorable polar solvation contribution (Δ*G*_GB_), which results in the instability of the Mol 3 complex. These results are expected to provide some useful information to design potential B-Raf inhibitors.

## Supplementary Materials

Supplementary materials can be found at http://www.mdpi.com/1422-0067/16/11/26026/s1.
