# Supplementary material for: An Investigation of Molecular Docking and Molecular Dynamic Simulation on Imidazopyridines as B-Raf Kinase Inhibitors
Source: Int J Mol Sci. 2015 Nov 16;16(11):27350–61. doi: 10.3390/ijms161126026 (PMC4661884; doi:10.3390/ijms161126026)
Supplement: Supplementary file 1 [file ijms-16-26026-s001.pdf]

Supplementary Materials: An Investigation of Molecular Docking and Molecular Dynamic Simulation on Imidazopyridines as B-Raf Kinase Inhibitors

Huiding Xie, Yupeng Li, Fang Yu, Xiaoguang Xie, Kaixiong Qiu and Jijun Fu

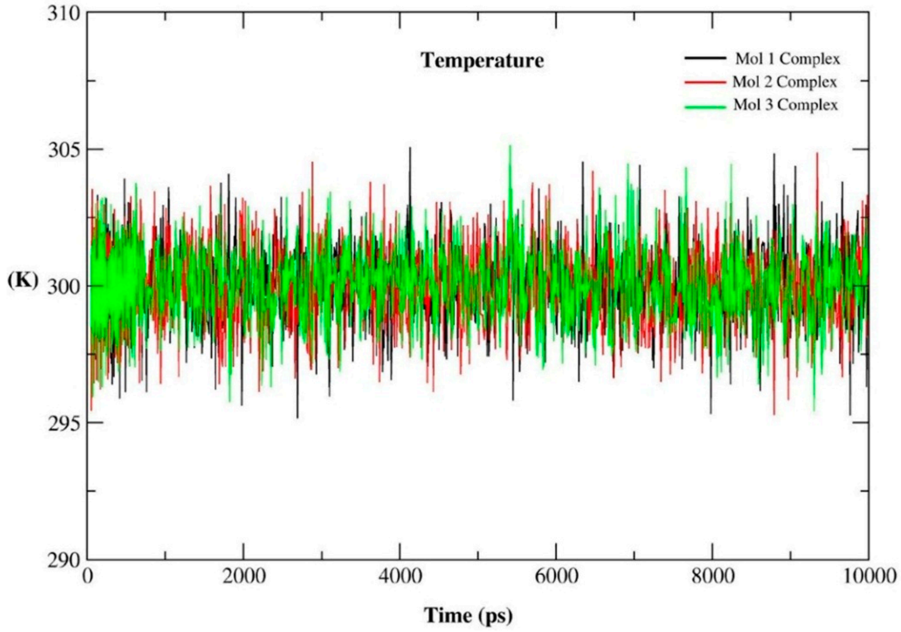

Figure S1. The temperature fluctuation *versus* time.

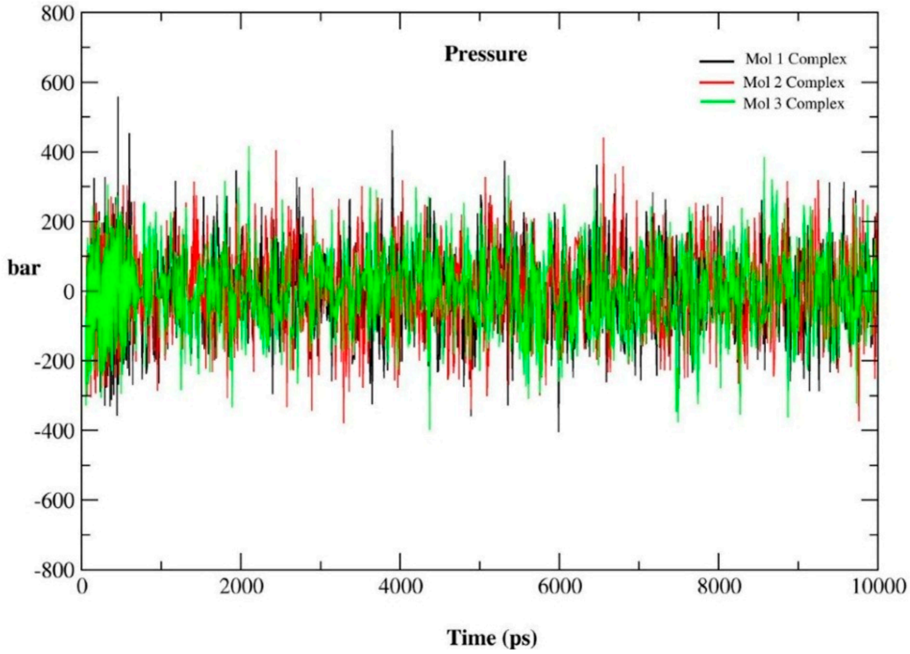

Figure S2. The pressure fluctuation *versus* time.

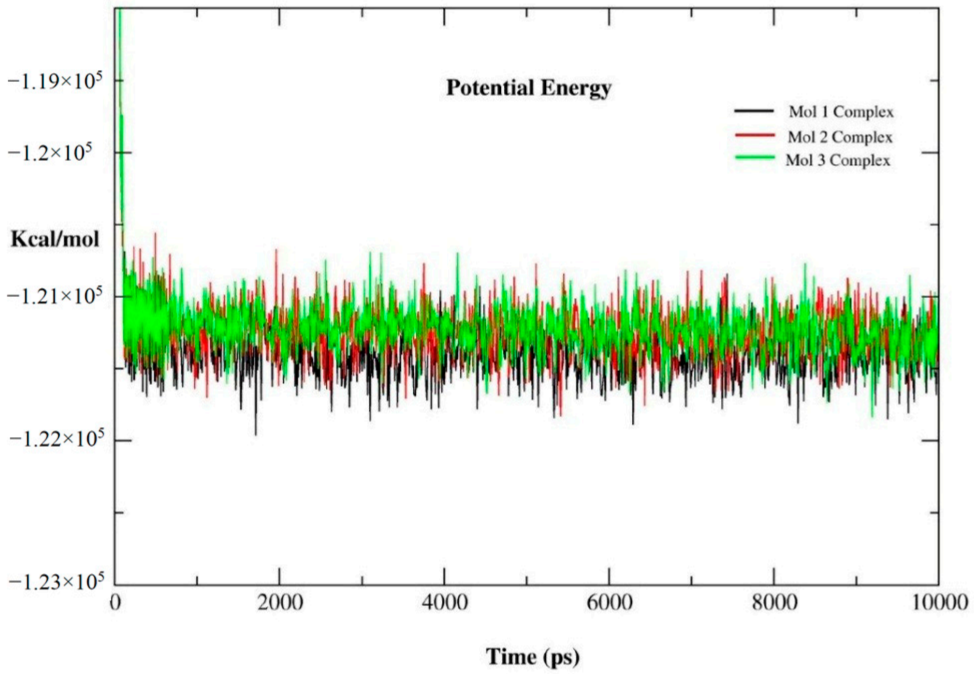

Figure S3. The potential energy fluctuation *versus* time.

Table S1. Chemical structures, biological activity values, and docking C_scores of the imidazopyridines.

Compound	General Structure	Substituent	IC_{50} (nM)	pIC_{50}	C_Score
**1** (Mol 1)		H	61	7.215	6.37
**2**		Me	40	7.398	7.27
**3**		Et	59	7.229	5.97
**4**	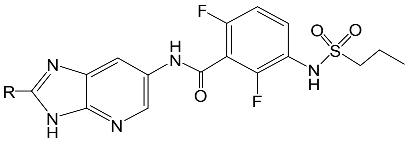	*i*-Pr	60	7.222	7.13
**5**		*t*-Bu	69	7.161	7.07
**6**		Cyclobutyl	31	7.509	5.98
**7**		4-Piperidine	107	6.971	7.22
**8** (Mol 3)		3-Piperidine	167	6.777	7.56
**9**		H	3.6	8.444	7.62
**10**		4-F	4.4	8.357	9.57
**11**		4-Cl	2.2	8.658	9.56
**12**		4-Br	2.2	8.658	9.48
**13**		3-F	3.1	8.509	9.53
**14**		3-Cl	1.1	8.959	9.71
**15** (Mol 2)		3-Br	0.76	9.119	9.74
**16**		2-F	8.0	8.097	9.20
**17**		2-Cl	27	7.569	7.38
**18**		2-Br	27	7.569	7.42
**19**	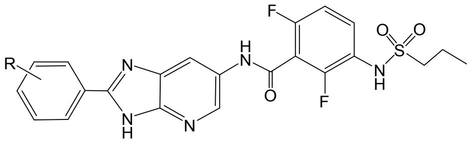	3,4-di-F	3.4	8.469	8.32
**20**		3,4-di-Cl	1.5	8.824	8.54
**21**		4-MeO	1.1	8.959	7.85
**22**		4-Me	1.3	8.886	9.51
**23**		4-CF_3	1.4	8.854	8.25
**24**		4-CF_3O	2.4	8.62	7.95
**25**		4-CN	2.7	8.569	8.18
**26**		4-$MeSO_2$	1.4	8.854	8.01
**27**		3-MeO	1.2	8.921	9.42
**28**		3-CF_3	1.0	9.000	8.79
**29**		4-Pyridyl	3.2	8.495	7.74
**30**		3-Pyridyl	3.0	8.523	9.39
**31**		H	1.0	9.000	8.19
**32**	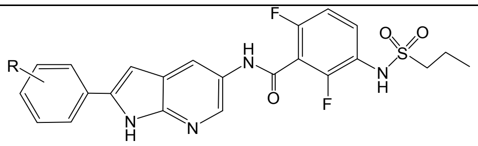	4-F	1.1	8.959	6.71
**33**		4-Cl	2.4	8.62	8.38
**34**		H	4.6	8.337	6.13
**35**	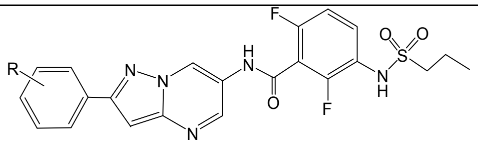	4-F	11	7.959	4.77
**36**		4-Cl	8.2	8.086	5.30
